# *PRKACB* is a novel imprinted gene in marsupials

**DOI:** 10.1186/s13072-024-00552-8

**Published:** 2024-09-28

**Authors:** Trent Newman, Donna M. Bond, Teruhito Ishihara, Phoebe Rizzoli, Quentin Gouil, Timothy A. Hore, Geoff Shaw, Marilyn B. Renfree

**Affiliations:** 1 School of BioSciences, The University of Melbourne, Melbourne, VIC 3010 Australia; 2 Department of Anatomy, University of Otago, Dunedin, New Zealand; 3 Epigenetics and Development Division, Walter and Eliza Hall Institute of Medical Research, Melbourne, VIC 3010 Australia; 4 Present Address: Epigenetics Programme, Babraham Institute, Cambridge, CB22 3AT UK

**Keywords:** Genomic imprinting, *PRKACB*, *GNAS*, Convergent evolution, Differential methylation

## Abstract

**Background:**

Genomic imprinting results in parent-of-origin-specific gene expression and, among vertebrates, is found only in therian mammals: marsupials and eutherians. A differentially methylated region (DMR), in which the methylation status of CpG dinucleotides differs between the two alleles, can mark the parental identity of imprinted genes. We developed a computational pipeline that detected CpG islands (CGIs) marked by both methylated and unmethylated signals in whole genome bisulfite sequencing data. This approach identified candidate marsupial DMRs in a publicly available koala methylome. One of these candidate DMRs was associated with *PRKACB*, a gene encoding the protein kinase A catalytic subunit beta. Nothing is known about the imprinting status of *PRKACB* in eutherian mammals although mutations of this gene are associated with endocrine neoplasia and other developmental disorders.

**Results:**

In the tammar wallaby and brushtail possum there was parent-of-origin-specific DNA methylation in the *PRKACB* DMR in which the maternal allele was methylated and the paternal allele was unmethylated. There were multiple RNAs transcribed from this locus. Allele-specific expression analysis identified paternal expression of a *PRKACB* lncRNA and an mRNA isoform. Comparison of the *PRKACB* gene start site between marsupials and eutherians demonstrated that the CGI is longer in marsupials. The *PRKACB* gene product functions in the same signalling pathway as the guanine nucleotide-binding protein alpha subunit encoded at the *GNAS* locus, a known eutherian imprinted gene. In a mouse methylome *Gnas* had three differentially methylated CGIs, while in the koala methylome the *GNAS* locus had two unmethylated CGIs.

**Conclusions:**

We conclude that *PRKACB* is a novel, DMR-associated marsupial imprinted gene. Imprinting of *PRKACB* in marsupials and *GNAS* in eutherians may indicate a conserved selection pressure for imprinting of the protein kinase A signalling pathway in therians with the two lineages adapting by imprinting different genes.

**Supplementary Information:**

The online version contains supplementary material available at 10.1186/s13072-024-00552-8.

## Introduction

Genomic imprinting is an epigenetic phenomenon that among vertebrates has so far only been detected in eutherian and marsupial mammals [3, 17, 75, 78]. Imprinting has predominantly been studied in eutherians, particularly mice and humans which each have a set of more than 200 known imprinted genes with 63 of these imprinted genes common to both species [59, 104]. Genomic imprinting has mainly been characterised in the placenta, contributing to an emphasis on roles for imprinted genes in fetal growth and the acquisition of maternal resources [26, 29, 57, 77, 94]. A broader range of functions for imprinted genes is also recognised in the development and physiology of the brain, mammary gland, immune system, and circadian system [29, 72, 95, 104].

Marsupials differ from eutherians primarily in their mode of reproduction [78]. Pregnancy in marsupials is relatively short and is supported in most species by a choriovitelline (yolk sac) placenta [21, 105]. Marsupials give birth to highly altricial young that are nourished, usually in a pouch (pouch young: PY), by a sophisticated lactation throughout their extended post-natal developmental period [25, 77, 103]. Genomic imprinting in marsupials is under-explored given that imprinting of genes in the context of a different reproductive strategy could provide insights into the evolution of imprinting. Imprinting of genes has been studied in two American marsupials: the grey short-tailed opossum [10, 14, 80] and the Virginia opossum [42, 109], and two Australian marsupials: the tammar wallaby [79, 98] and the brushtail possum [8].

So far, 25 autosomal genes are known that have parent-of-origin-specific gene expression in marsupials [8, 10, 14, 24, 35, 63, 90, 98, 100]. Marsupials have imprinting of orthologues of several known eutherian imprinted genes in placenta and in fetal tissues [77], post-natal tissues [95] and putative marsupial-specific imprints [10, 14]. Seven differentially methylated regions (DMRs) have been confirmed on marsupial autosomes (Table 1). Many DMRs are proximal to an imprinted long noncoding RNA (lncRNA) and a cluster of associated genes that have parent-of-origin-specific expression, as is the case at the *H19* locus and the insulin-like growth factor 2 receptor, *IGF2R,* locus [35, 90, 100].

**Table 1:** Known marsupial DMRs

Allele methylated	Gene symbol	Gene name	Species	Citation
Paternal	*GPX7*	Glutathione peroxidase 7	Brushtail possum	Bond et al. [8]
Paternal	*H19*; *IGF2*	Noncoding RNA H19; Insulin like growth factor 2	Tammar wallaby	Smits et al. [90]
Maternal	*IGF2R*; *ALID*	Insulin like growth factor 2 receptor; Antisense lncRNA in the *IGF2R* DMR	Tammar and grey short-tailed opossum	Das et al. [13] and Suzuki et al. [100]
Maternal	*MLH1*; *EPM2AIP1*	MutL homolog 1; Epilepsy progressive myoclonus type 2A interacting protein 1	Brushtail possum	Bond et al. [8]
Maternal	*NPDC1*; *POU5F3*	Neural proliferation differentiation and control protein 1; POU domain class 5 transcription factor 3	Grey short-tailed opossum	Cao et al. [10]
Maternal	*PEG10*	Paternally expressed gene 10	Tammar	Suzuki et al. [97]
Maternal	*PRKACB*	Protein kinase cAMP-activated catalytic subunit beta	Tammar	Present study
Maternal	*UBP1*	Upstream binding protein 1	Brushtail possum	Bond et al. [8]

Marsupial DMRs confirmed by allele-specific methylation analysis. In rows where more than one gene symbol and gene name is noted the DMR is associated with more than one proximal gene

Of particular interest are orthologous genes present in both eutherians and marsupials that are only imprinted in marsupials. The myeloid ecotropic viral integration site homeobox 1, *MEIS1*, gene was the first gene identified to be imprinted in marsupials (by transcriptionally opposing histone modifications in the grey short-tailed opossum) but not eutherians, though imprinting in eutherians was not formally tested [14]. A maternally-methylated DMR was identified in the opossum at the start site of the neural proliferation, differentiation and control 1 gene and the POU domain class 5 transcription factor 3 (*NPDC1* and *POU5F3*)*,* two paternally-expressed genes that share their first two exons [10]. The *NPDC1* and *POU5F3* genes have not been reported to be imprinted in any eutherian species suggesting these genes form a novel marsupial-specific imprinting cluster [10]. Bond et al. [8] identified four more novel marsupial-specific DMRs at glutathione peroxidase 7 (*GPX7*), the mutL homolog 1 gene and epilepsy progressive myoclonus type 2A interacting protein 1 (*MLH1* and *EPM2AIP1*) and upstream binding protein 1 (*UBP1*), demonstrating that there is a unique suite of marsupial imprinted genes. They suggested these evolved in concert with the specific developmental and reproductive traits characteristic of this mammalian lineage [8].

To find previously unknown imprinted genes in marsupials we identified candidate DMRs in a publicly available koala brain methylome [88]. Whole genome bisulfite sequencing (WGBS) has been a common way to detect DNA methylation based on bisulfite conversion of unmethylated cytosines [22, 50]. In the present study, a candidate DMR from the koala WGBS data located near the gene *PRKACB* (protein kinase cAMP-activated catalytic subunit beta) was assessed further. Nothing has been reported about the imprinting status of *PRKACB* in any eutherian species. In a complementary study, *PRKACB* featured on a shortlist of candidate imprinted genes based on allele-specific methylation in a single brushtail possum individual and evidence for monoallelic expression [8].

The *PRKACB* gene encodes a catalytic subunit of protein kinase A (PKA). The PKA pathway, also known as the cAMP (cyclic adenosine 3′, 5′-monophosphate) pathway, is a well-studied model of cellular signal transduction [102]. Activation of G protein-coupled receptors (GPCRs) by extracellular signaling molecules at the cell membrane leads to downstream activation of adenylyl cyclase, via the G-protein alpha-subunit (G_s_α), and synthesis of the secondary messenger cAMP [83]. PKA is an enzyme consisting of two cAMP-binding regulatory (R) subunits and two catalytic (C) subunits [93]. Binding of cAMP to the regulatory units of PKA releases the catalytic subunits to phosphorylate various target substrates in the cytoplasm and nucleus [93].

The two major PKA C subunit genes are *PRKACA* and *PRKACB*, with more known about *PRKACA* [102]. Three additional C subunit genes have been identified in humans: *PRKX* and the retrotransposon-derived genes *PRKY* and *PRKACG* [76, 102]. The PKA C subunits have different affinities for certain peptide substrates, suggesting that the signalling downstream of PRKACB differs from that of PRKACA [23, 91, 93]. A well-known substrate of PKA-mediated phosphorylation is the cAMP response element binding protein (CREB) transcription factor which regulates the expression of approximately 4000 genes that have various functions in cell metabolism, the cell cycle and cellular secretory pathways [83, 110].

Altered PRKACB function has been implicated in various aspects of human health and disease. Somatic mutation near the PRKACB active site, p.Ser54Leu, led to adrenal tumor in a patient with severe Cushing syndome [19]. Triplication of a 1.6-mb region of chromosome 1p31.1, including *PRKACB*, resulted in a case of Carney complex presenting with acromegaly, pigmented spots, and myxomas [20]. A patient with multiple skeletal developmental malformations had a possible pathogenic mutation of PRKACB, p.Lys286del, in a position that could interfere with PKA R subunit binding [18]. In four unrelated patients, mutation near the PRKACB active site, p.Ser54Leu and p.His88Arg, or the protein partner tethering surface, p.Gly235Arg, caused congenital heart abnormalities and polydactyly [68]. Translocation and gene fusion between *PRKACB* and ATPase Na^+^/K+ transporting subunit beta 1 (*ATP1B1*) resulted in papillary neoplasms of the pancreas and bile duct [89].

*PRKACB* functions in the same pathway as guanine nucleotide-binding protein alpha subunit (*GNAS*): a known imprinted gene in eutherians. Multiple products are generated from the complicated *GNAS* locus [12], including *G*_*s*_*α*, extra-long alpha subunit (*XLαs*), neuroendocrine secretory protein 55 (*NESP55*) and neuroendocrine secretory protein antisense (*NESPAS*). G_s_α functions upstream of PRKACB in the cAMP/PKA signaling pathway [83], though PRKACB can also activate G_s_α [37]. Parent-of-origin-specific expression from the *GNAS* locus is transcript- and tissue-specific, *G*_*s*_*α* is maternally-expressed in the adult pituitary and loss of *G*_*s*_*α* imprinting is associated with endocrine tumours [31]. Comparative analysis of the CpG content of the orthologous *GNAS* regions suggests imprinting of the *NESP* CpG island (CGI) in the *GNAS* domain has eutherian origins [17, 99].

Since marsupial-specific genomic imprinting could provide insights into the evolution of imprinting we asked whether new imprinted genes might be found in a publicly available marsupial methylome. Here we identified *PRKACB* as a candidate DMR-associated imprinted gene in the koala and find in the tammar and brushtail possum that the *PRKACB* DMR was maternally-methylated and associated with expression from the paternal allele. Comparison of the *PRKACB* start site between species showed that the CGI is longer in marsupials than eutherians. We conclude that *PRKACB* is an imprinted gene in marsupials and potentially a marsupial-specific imprinted gene.

## Results

### Identification of new marsupial DMR candidates

**Fig. 1:**
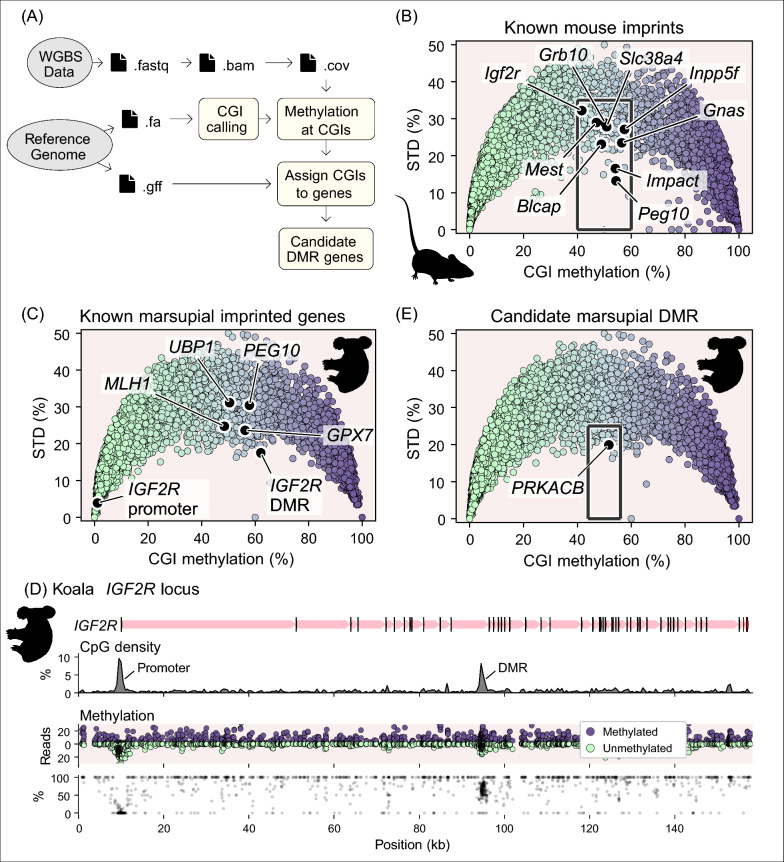
Pipeline for candidate DMR detection identifies *PRKACB*. **A** Schematic of the processes involved in the bioinformatic DMR candidate detection pipeline. The steps indicated by the yellow boxes were achieved using custom scripts. **B**, **C**, **E** CGIs were assessed for methylation in terms of the percent of WGBS reads that were methylated and the standard deviation (STD) of the methylation across the CpG sites in the CGI. **B** Known mouse imprinted genes have differentially methylated CGIs in a mouse brain methylome. **C** Known marsupial imprinted genes had differentially methylated CGIs in a koala brain methylome, the koala methylome highlighted an unmethylated *IGF2R* promoter CGI and a differentially methylated intragenic CGI. **D** Illustration of the koala *IGF2R* locus, exons are arrowheads indicating the direction of transcription, introns are thinner shaded regions (red indicates maternal expression). CpG density is plotted as a percentage averaged over 500 bp windows. Methylated (purple) and unmethylated (green) WGBS read counts and the percent methylation are plotted over the region. **E** Grey boxes indicate the selection gates for koala DMR candidates, the *PRKACB* candidate DMR is indicated. Note, plots C and E contain the same data but highlight different genes of interest. Species silhouettes are *Murinae* and *Phascolarctos* *cinereus*, courtesy of PhyloPic.org

A pipeline was set up to detect candidate marsupial DMRs. To assess the methylation status of CGIs a publicly available koala brain WGBS dataset [88] and the annotated koala reference genome (phaCin_unsw_v4.1: [39]) were used as inputs (Fig. 1A). This approach detected 17,365 CGIs (Fig. 1C), 12.0% of these CGIs were highly methylated (> 80% methylated reads) and 77.7% were highly unmethylated (< 20% methylated reads). CGIs of interest as candidate DMRs were those that possessed a combination of methylated and unmethylated reads.

We tested the ability of this pipeline to identify known imprinted genes. In a mouse brain WGBS data set (GSM1173783) known imprinted genes, including *Gnas*, growth factor receptor bound protein 10 (*Grb10*), *Igf2r*, and paternally expressed gene 10 (*Peg10*), were found to have a CGI with a methylation level between 40 and 60% (Fig. 1B). The pipeline was also tested by examining the marsupial *IGF2R* locus as the tammar *IGF2R* gene is known to have an unmethylated promoter CGI and a maternally-methylated intragenic DMR [100]. The koala brain WGBS data gave two distinct CGI methylation signals for *IGF2R* (Fig. 1C, D), the promoter CGI was 0.8% methylated and the intragenic CGI was 64.3% methylated. Other known marsupial DMRs were detected including *MLH1*, *UBP1*, *GPX7* and *PEG10* (Fig. 1C). This pipeline did not detect the known DMRs at *NPDC1* and *H19*. The *NPDC1* DMR was relatively short with a low overall CpG percentage. The *H19* DMR was relatively long and highly variable in the CpG percentage.

**Fig. 2:**
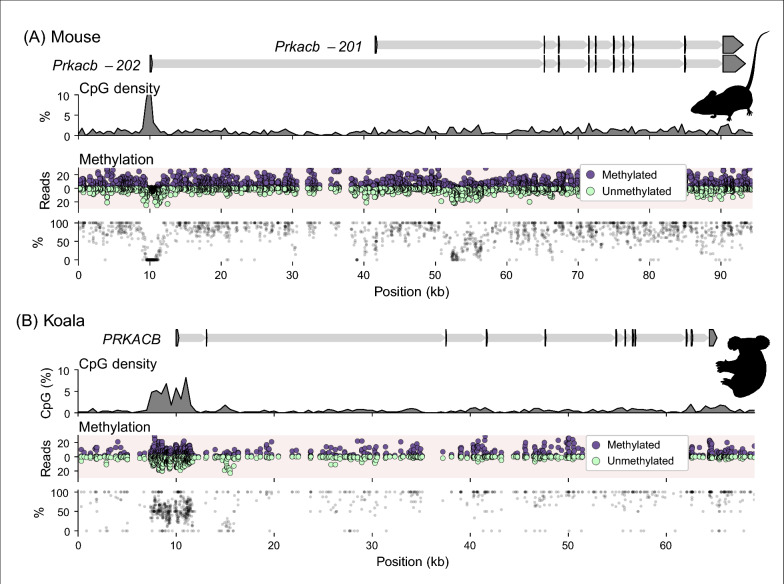
Differential methylation of koala but not mouse *PRKACB* CGI. Scale illustration of the **A** mouse *Prkacb* locus and the **B** koala *PRKACB* locus, exons are arrowheads indicating the direction of transcription, introns are thinner shaded regions. The structures of two *Prkacb* isoforms are indicated for the mouse. CpG density is plotted as a percentage averaged over 500 bp windows. Methylated (purple) and unmethylated (green) WGBS read counts and the percent methylation are plotted over the region. Species silhouettes are *Mus musculus* and *Phascolarctos* cinereus, courtesy of PhyloPic.org

Applying a stringent criteria to enrich for compelling CGIs (see Methods section) produced a shortlist of candidate DMRs. Of these candidates we focused on *PRKACB* (Fig. 1E) as it already featured in a shortlist of candidate imprinted genes from the brushtail possum with allele-specific methylation in one individual with evidence for mono-allelic expression albeit with parental origin unknown [8]. The *PRKACB* gene had a CGI with a methylation level of 51.7% in the koala WGBS data but was unmethylated in the mouse WGBS data (Fig. 2). The mixed methylation signal was associated with a prominent CGI at the gene start site that had a CpG density of 4.7% over a 4 kb region (Fig. 2B).

### The marsupial *PRKACB* CGI is methylated on the maternal allele

**Fig. 3:**
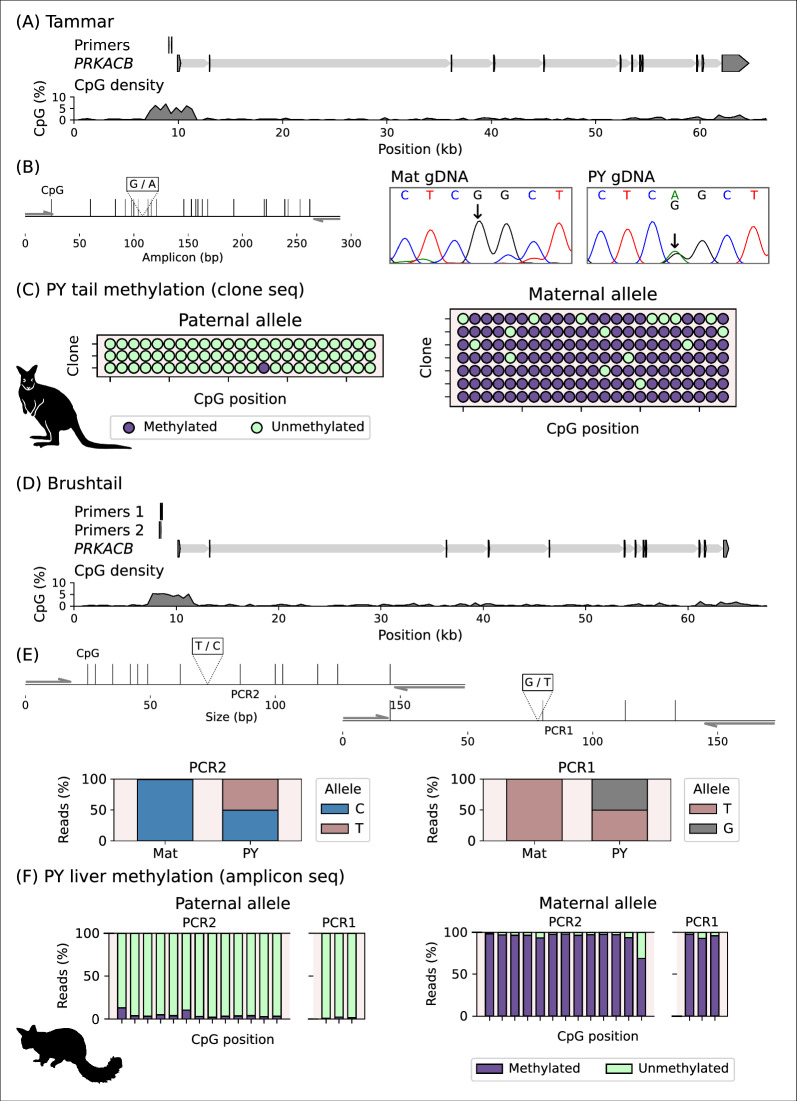
Differential methylation of marsupial *PRKACB* CGI. **A** The *PRKACB* locus in tammar, the position of the bisulfite primers used to clone the CGI region for DNA methylation analysis are indicated. **B** The position of the CpG sites and SNP within the assessed amplicon. Sanger genotyping of the SNP used for allele-phasing of methylation signals. **C** Allele-specific methylation analysis of tammar tail tissue showing methylated (purple) and unmethylated (green) CpG sites on the paternal (left) and maternal (right) allele for one of four animals. **D** The *PRKACB* locus in brushtail possum, the position of the bisulfite primers used for DNA methylation by amplicon sequencing. **E** The position of the CpG sites and SNPs within the two assessed amplicons. Illumina genotyping of the SNPs used for allelic-phasing of methylation signals in PCR amplicons 1 and 2. **F** Allele-specific methylation analysis in the brushtail possum liver showing methylated (purple) and unmethylated (green) reads at 15 CpG sites (assessed by 2 bisulfite amplicons) on the paternal (left) and maternal (right) allele for one of two animals. Species silhouettes are *Phascolarctos cinereus*, *Notamacropus (Macropus) eugenii* and *Trichosurus vulpecula*, courtesy of PhyloPic.org

To test whether the mixed signal of methylated and unmethylated reads detected at the *PRKACB* CGI in the koala WGBS data was the result of parent-of-origin-specific methylation, the orthologous region was assessed by a targeted locus-specific analysis in the tammar wallaby and brushtail possum. Like in the koala, the *PRKACB* locus in the tammar and brushtail possum had a CpG-rich region present at the gene start site (Fig. 3).

Allele-specific phasing of bisulfite-converted DNA from the tammar and brushtail possum separated out the methylated DNA sequences by their parent of origin. Genotyping 11 tammar PY showed five animals with SNPs in the candidate DMR region (Fig. 3A, B), four of these animals had informative homozygous mothers. Of 23 CpG sites assessed in tammar PY tail tissue by bisulfite clone sequencing (Fig. 3C) the maternal DNA was 90.7% methylated (15 total clones, 4 animals) while the paternal DNA was 0.3% methylated (14 total clones, 4 animals). Genotyping five brushtail possum PY showed two animals with SNPs, in either one or both bisulfite amplicons targeting within in the candidate DMR region (Fig. 3D, E), that had informative homozygous mothers. Allele-specific methylation in the brushtail possum was assessed in two adjacent regions by bisulfite amplicon sequencing (Fig. 3F). Of the three CpG sites assessed in one bisulfite amplicon, the maternal DNA from the 2 animals was 95.3% and 94.7% methylated (122 and 449 reads) while the paternal DNA was 1.6% and 2.6% methylated (128 and 441 reads), respectively. A second amplicon with 13 CpG sites, showed the maternal DNA from the 1 animal was 94.4% methylated (281 reads) while the paternal DNA was 4.6% methylated (442 reads).

### Multiple transcripts produced from the marsupial *PRKACB* locus

To understand how differential methylation might regulate *PRKACB*, we assessed which RNAs were transcribed from the locus.

**Fig. 4:**
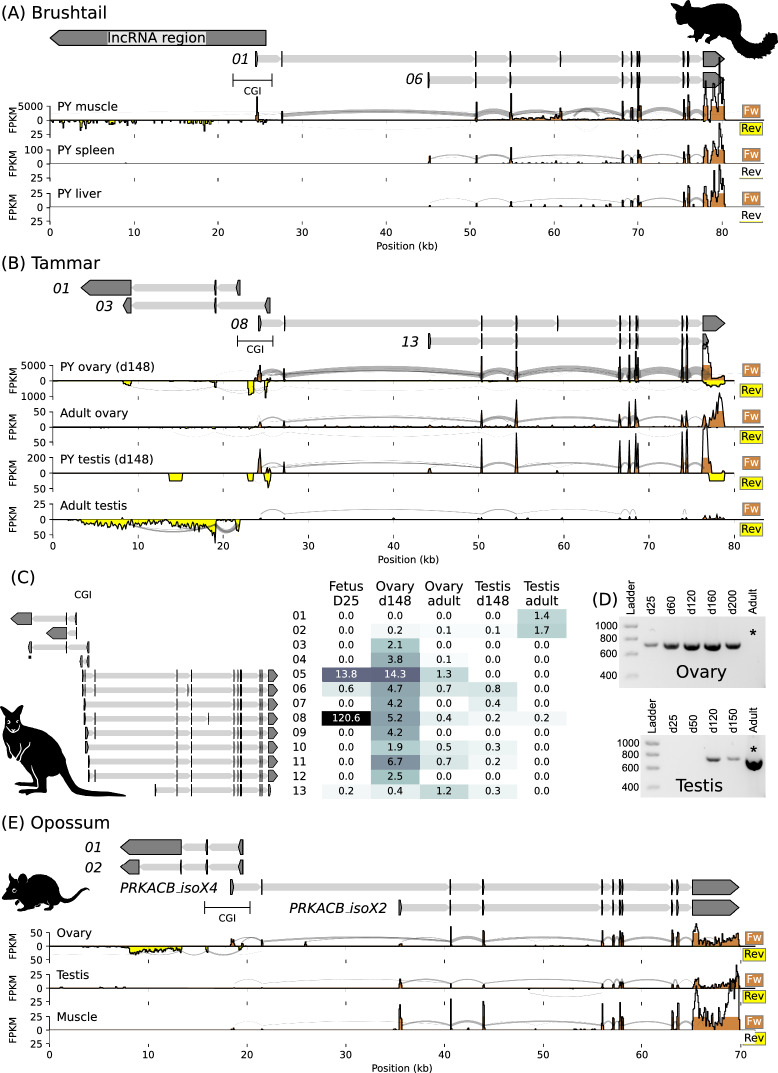
Transcription from the *PRKACB* locus. **A** The brushtail possum *PRKACB* locus illustrating selected transcript structures detected in short read transcriptomes from PY muscle, spleen and liver and the estimated region containing a low-level lncRNA signal in muscle. Below, the coverage tracks of forward (brown) and reverse (yellow) transcript mapped reads for the different tissues, “sashimi” lines reflect junction-spanning reads with the width of the lines a function of the number of reads. **B** The tammar *PRKACB* locus illustrating selected transcript structures detected across short read adult gonad transcriptomes and long read transcriptomes from a term fetus and PY gonads. **C** The structure, compressed horizontally relative to **B**, and expression level of all of the merged Stringtie transcript stuctures in the tammar. The expression level for each transcript is reported as FPKM, and given a colour to reflect this value, for the different tammar tissues. **D** RT-PCR gels showing the presence of the lncRNA at different stages of ovary (top) and testis (bottom) development. Asterisks highlight the difference between the ovary and testis in terms of expression at the adult stage. The position of the amplicon is indicated in **C**. **E** The opossum *PRKACB* locus illustrating selected transcript structures and annotated transcripts detected in short read transcriptomes from ovary, testis and muscle. Species silhouettes are *Trichosurus vulpecula*, *Notamacropus (Macropus) eugenii* and *Monodelphis domestica*, courtesy of PhyloPic.org

In the brushtail possum, six distinct *PRKACB* transcripts were detected by Stringtie (Fig. 4A) across the publicly available PY tissue transcriptomes [8] that were assessed (muscle, spleen and liver, and skin, not shown). The first five transcripts, found in the muscle and skin but not the liver and spleen, varied by exon use and had transcriptional start sites proximal to the DMR. Transcript “*01*” was the most abundant in muscle with 337 fragments per kilobase of transcript per million mapped reads (FPKM). *PRKACB* transcript “*06*” started 20.6 kb downstream of transcript “*01*” and was found in the liver and spleen at 1.3 and 6.3 FPKM, expression in the muscle was barely detected at 0.4 FPKM. Upstream of the brushtail possum DMR a transcription signal was observed from the reverse strand in the muscle tissue, indicative of an antisense transcript, but the expression level was too low to assemble a transcript structure so the “lncRNA region” is indicated (Fig. 4A).

Analysis of tammar transcriptomes resulted in the identification of four antisense (“*01*” to “*04*”) and nine sense transcripts (“*05*” to “*13*”) from the *PRKACB* locus (Fig. 4B, C). Long-read transcriptomes from a term fetus and PY (d148) gonads and publicly available short-read transcriptomes for adult gonads were assessed. The structure of the sense transcripts in the tammar resembled those found in the brushtail possum. Tammar *PRKACB* transcripts “*05*” to “*12*” had a start site proximal to the DMR and variation in exon use with small changes in the position of the transcriptional start site. Transcript “*08*” was the most abundant of these and was detected in all tissues, though at a low level in the PY and adult testis and had 120.6 FPKM in the D25 fetus (Fig. 4C). *PRKACB* transcript “*13*” (similar to brushtail possum transcript “*06*”) started 20.0 kb downstream of “*08*” and had 1.2 FPKM in the adult ovary and was not detected in the adult testis transcriptome.

Four distinct antisense long non-coding RNAs (lncRNAs), all with start sites within the DMR, were detected in the tammar transcriptomes (Fig. 4B, C). The reverse reads at the 5′ end of the *PRKACB* gene indicated antisense transcription of these lncRNAs. The low-level reverse reads at the 3′ end of the *PRKACB* gene in the long-read data were considered an artifact of an imperfect sense assignment method but could reflect another lncRNA. There appeared to be ovary and testis-specific transcription start site usage for lncRNA expression. Transcript “*01*” was expressed only in the short-read adult testis transcriptome with 1.4 FPKM and presented as a noisy signal in combination with a signal from transcript “*02*”*.* Transcript “*03*” differed from “*01*” in the 5′ exon and the length of the 3′ exon and presented as a clean signal with 2.1 FPKM in the long-read PY ovary transcriptome. RT-PCR using primers located in the common region of the 3′ exon of transcript “*01*” and “*03*” produced amplicons from PY ovaries, and adult testis, but not adult ovaries (Fig. 4D).

Upstream of the grey short-tailed opossum *PRKACB* CGI an lncRNA transcription signal was observed from the reverse strand in a publicly available ovary transcriptome (Fig. 4E). The structure of lncRNA transcripts detected by Stringtie, “*01*” and “*02*”, resembled a combination of the 5′ structures of the tammar “*01*” and “*03*” transcripts. Transcript “*01*” differed from “*02*” in the 3′ structure of the transcript, “*02*” had 1.5 FPKM and “*02*” had 0.2 FPKM. No transcription was observed from the reverse strand upstream of the *PRKACB* CGI in publicly available opossum testis and muscle transcriptomes.

### Allele-specific expression from the *PRKACB* locus

**Fig. 5:**
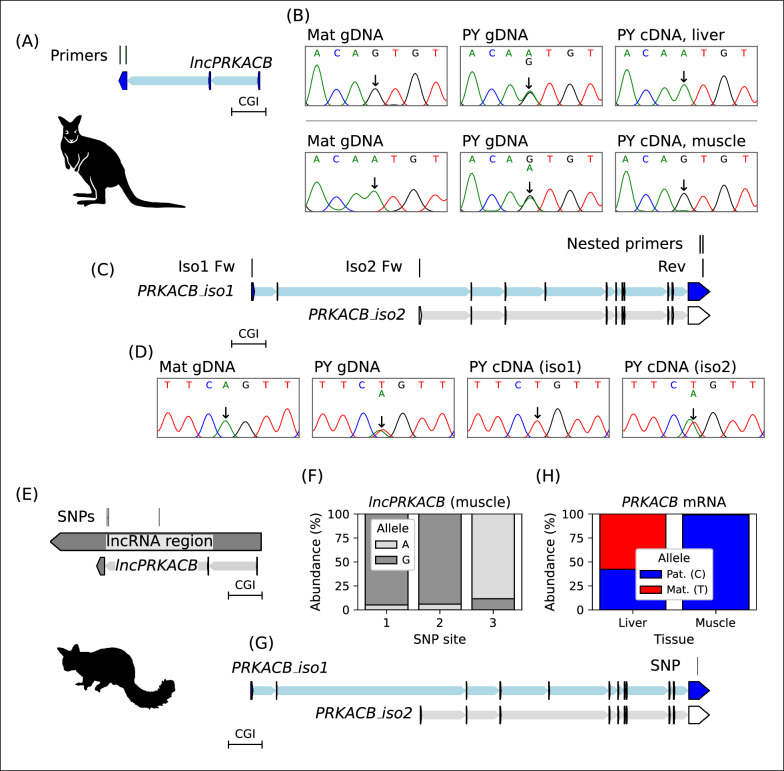
Allele-specific expression from the *PRKACB* locus. **A** The tammar *lncPRKACB* locus indicating the location of the primers used for allele-specific expression analysis. The blue transcript colour indicates paternal expression, the position of the CGI is provided for reference. **B** Allele-specific expression of *lncPRKACB* in tammar liver (top) and muscle (bottom), the horizontal line distinguishes two sets of mother-young. Informative homozygous genotype in a matched maternal sample (left), heterozygous SNP in PY genomic DNA (centre) and expression of *lncPRKACB* from the paternal allele (right). **C** The rest of the tammar *PRKACB* locus indicating coding transcripts, *PRKACB_iso1* and *PRKACB_iso2.* The location of isoform-specific and nested primers used for allele-specific expression is indicated. **D** Allele-specific expression of *PRKACB* is transcript-specific in tammar. The blue transcript colour indicates paternal expression, white transcript colour indicates biallelic expression. Informative homozygous genotype in a matched maternal sample (left), heterozygous SNP in PY genomic DNA (centre left) and expression of *PRKACB_iso1* (centre right) and *PRKACB_iso2* (right). **E** The brushtail possum *lncPRKACB* locus indicating the location of the SNPs used for allele-specific expression analysis. The exon positions are aligned from the tammar *lncPRKACB*. The grey transcript colour indicates unknown parent-of-origin-specific expression, the position of the CGI is provided for reference. **F** Monoallelic expression of the *lncPRKACB* across the three SNP sites in amplicon sequencing from brushtail possum PY muscle tissue. **G** The rest of the brushtail possum *PRKACB* locus indicating coding transcripts, *PRKACB_iso1* and *PRKACB_iso2.* The blue transcript colour indicates paternal expression, white transcript colour indicates biallelic expression. The location of the SNP used for allele-specific expression in the shared 3′ region is indicated. **F** Biallelic expression of *PRKACB* in a brushtail possum PY liver and monoallelic paternal expression in PY muscle

To test whether the *PRKACB* DMR was associated with parent-of-origin-specific gene expression we examined *PRKACB* expression in the tammar and brushtail possum. At least eleven tammar PY were genotyped at each of five locations throughout the *PRKACB* gene. The only locations where SNPs were found were the final exons of both the sense and antisense transcripts (Fig. 5A). We first focused on antisense transcription as a target for allele-specific expression in the tammar because the 3′ exon from *lncPRKACB* frequently contained allelic variation. Transcript “*03*” was 1,271 base pairs (bp) long and the Coding Potential Calculator 2 [41] classified it as a noncoding sequence with a low coding probability of 0.01. We called transcripts “*01*” and “*03*” as *lncPRKACB.* Allele-specific expression of *lncPRKACB* was tested using seven SNP sites detected in the 3′ exon. Four PY were identified that had an informative maternal genotype (Fig. 5B), RT-PCR produced an amplicon for the *lncPRKACB* from the PY liver which had monoallelic expression from the paternal allele in all PY sampled. A further six PY, for which muscle samples had been collected, were genotyped. Both PYs that had an informative maternal genotype had monoallelic paternal expression of *lncPRKACB* in the muscle tissue (Fig. 5B), the two PY without an informative maternal genotype also had monoallelic expression.

To test whether the *PRKACB* coding gene had allele-specific expression, two of the sense transcripts were considered (Fig. 5C). Transcript “*08*” was used to model *PRKACB* transcription from the DMR and was here called *PRKACB_iso1.* Transcript “*13*” was of interest due to its different start site and was here called *PRKACB_iso2.* Both isoforms had high coding probabilities > 0.99, *PRKACB_iso1* was 3,876 bp and was predicted to code for a peptide 387 amino acids (aa) long while *PRKACB_iso2* was 3,715 bp and had a predicted peptide length of 399 aa. No SNPs were detected in the exons unique to these transcripts, so a nested approach was taken where the specific transcript was first amplified by RT-PCR and then assessed for allele-specific expression using SNPs in the shared 3′ region of the gene with a second round of PCR (Fig. 5C).

Allele-specific expression of the *PRKACB* coding transcripts was isoform-specific. Of eleven animals genotyped three PYs had a SNP found in the shared 3′ region and only one of these animals had an informative homozygous maternal genotype though the results in this animal were corroborated by the brushtail possum, below. Amplification of *PRKACB_iso1* resulted in a single ~3 kb band and nested amplification of the shared 3′ region showed monoallelic expression from the paternal allele (Fig. 5D). RT-PCR for *PRKACB_iso2* resulted in multiple bands, nested allele-specific expression analysis of the expected ~3 kb product showed *PRKACB_iso2* to be biallelically-expressed (Fig. 5D).

Allele-specific expression from the lncRNA region of the *PRKACB* locus was also assessed in the brushtail possum. Genotyping within the lncRNA region identified three SNP sites (Fig. 5E) in a PY for which muscle tissue was available: the mother was heterozygous at these sites preventing parent-of-origin analysis. Transcription from the brushtail possum lncRNA region in this PY muscle was monoallelic (Fig. 5F) with expression of the minor allele for the three SNPs at 5.1%, 6% and 11.7% and the average major allele expression at 92.4% (as determined from a total of 2112, 3028 and 1121 amplicon reads, for each respective SNP). The first two SNPs in the brushtail possum were located in the region equivalent to the 3′ exon of the tammar “01” and “03” transcripts, the third SNP also had monoallelic expression despite being in the region equivalent to the 3′ exon of the tammar “02” transcript (Fig. 5E). No transcripts were detected in three brushtail possum PY liver samples using RT-PCR with the three lncRNA primer sets.

Allele-specific expression of the *PRKACB* coding gene was tested in the brushtail possum. Like in the tammar, two sense transcripts were considered; transcript “*01*” had a start site proximal to the DMR and was called *PRKACB_iso1*, transcript “*06*” with a different start site was called *PRKACB_iso2* (Fig. 5G). The transcriptome analysis (Fig. 4A) showed *PRKACB_iso1* predominantly expressed in the muscle while the liver expressed *PRKACB_iso2*. Five brushtail possum PY for which liver samples were available were genotyped and all had a SNP in the 3′ exon of the isoforms. Four PY had an informative maternal genotype. Expression of *PRKACB* (*PRKACB_iso2*) in the liver was biallelic (Fig. 5H) with the maternal allele present at 48.4% and the paternal allele present at 51.6% (average 1,163 ± 330 reads per animal, 4 animals). The two brushtail possum PY for which muscle samples were available both had a SNP and one of these PY had an informative maternal genotype. *PRKACB* (*PRKACB_iso1*) was monoallelically expressed from the paternal allele in the muscle (Fig. 5H) with the paternal allele present at 99.1% (25,753 total reads). The muscle sample for which the mother was heterozygous had monoallelic expression of the SNP with the major allele present at 99.8% (60,678 total reads).

### Marsupials have a longer CGI at the *PRKACB* start site

**Fig. 6:**
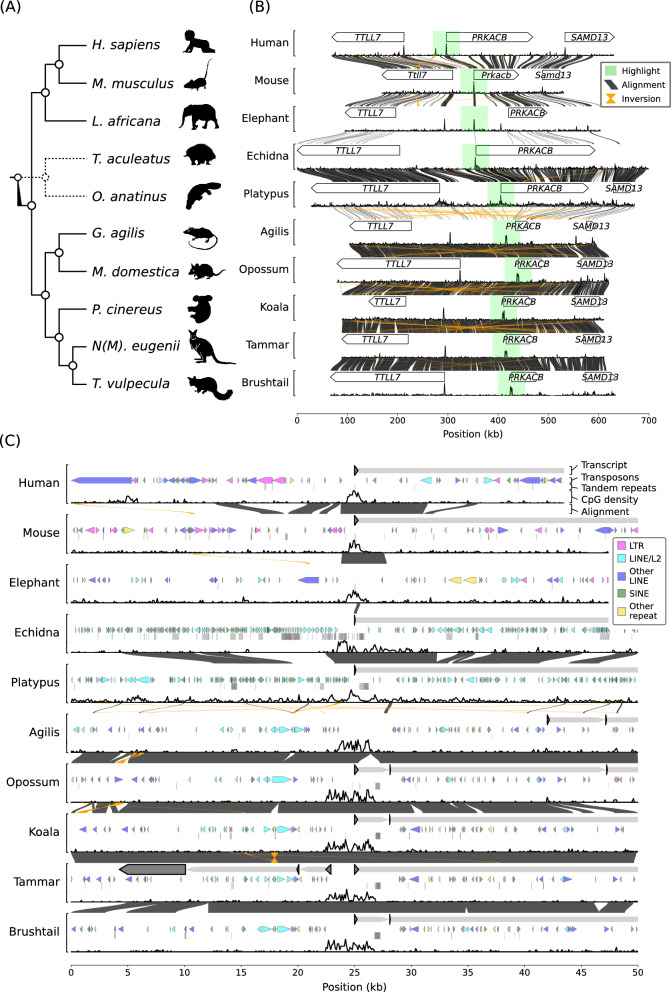
Marsupial-specific *PRKACB* CGI structure. **A** Cladogram indicating the phylogenetic relationship of the monotreme, marsupial and eutherian species analysed. Monotremes diverged before the separation of therians but are positioned (dotted line) between eutherians and marsupials to facilitate comparison. **B** Synteny comparison of the region containing the *TTLL7*, *PRKACB* and *SAMD13* genes. The annotated gene regions are shown as white arrowheads, CpG density is shown below, averaged over 500 bp windows. Dark grey links indicate pairwise alignments between two species, orange links indicate inverted alignments. The green shaded region highlights the *PRKACB* start site. **C** Repeat element composition and CpG density over the CGI region at the *PRKACB* start site of different animals. A 50 kb region is plotted for each animal, centered on the gene start site, with CpG density averaged over 100 bp windows. The location of the tammar *lncPRKACB* transcript is indicated. LTRs are pink, LINE/L2 elements are cyan, other LINEs are dark blue, SINEs are green and other transposons are yellow. Below the transposable elements, tandem repeats are indicated as grey lines. The mouse, elephant, platypus, agile gracile opossum, grey short-tailed opossum, brushtail possum regions were reversed to correct for larger inversions or a different chromosome orientation relative to the other species. Species silhouettes are *Homo sapiens*, *Mus musculus*, *Loxodonta africana*, *Tachyglossus aculeatus*, *Ornithorhynchus anatinus*, *Gracilinanus*, *Monodelphis domestica*, *Phascolarctos cinereus*, *Notamacropus (Macropus) eugenii* and *Trichosurus vulpecula*, courtesy of PhyloPic.org

The imprinting status of *PRKACB* has not been reported in any species, so we compared the *PRKACB* locus between monotremes, marsupials and eutherians (Fig. 6A). Gene synteny was conserved in mammals. In all the species assessed *PRKACB* was located between the tubulin tyrosine ligase like 7 (*TTLL7*) gene and the sterile alpha motif domain containing 13 (*SAMD13*) gene (Fig. 6B). The broader *PRKACB* region was similar within marsupials and within monotremes. There was pairwise alignment between 83.1% and 87.8% of the opossum and koala regions respectively. The broader *PRKACB* region showed more divergence within eutherians, there was pairwise alignment between 38% and 57.8% of the human and mouse regions.

A notable CGI was present at the annotated *PRKACB* start site in all of the species (Fig. 6). The shorter CGI in eutherians had a higher maximum CpG density of 19% in humans and 15% in mice while in marsupials the maximum CpG density was 11%. In mice (see also Fig. 2A) and humans the CGI spanned an ~1 kb region. The *PRKACB* CGI was longer in marsupials, the koala (see also Fig. 2B) and tammar CGI spanned ~4 kb. The *PRKACB* CGI was ~3 kb in the agile gracile opossum.

The *PRKACB* CGI was not closely related at the sequence level between the three groups of mammals (Fig. 6C). The elephant and echidna shared a small ~250 bp comparable segment at the first exon of *PRKACB*, the marsupial *PRKACB* CGI did not align well with the orthologous monotreme or eutherian sequences. Tandem repeats did not make a notable contribution to the length of the marsupial CGI in marsupials (with the exception of the agile gracile opossum) the DNA immediately 3′ adjacent to the CGI contained more than 120 copies of a “CCT” repeat. A “CCG” tandem repeat was present in the center of the eutherian CGI, in humans this was 54 bp long (18 copies). In the echidna there was a 736 bp segment comprised of tandem repeats located at a peak in CpG density at the *PRKACB* start site.

Few transposable elements were found within the *PRKACB* CGI (Fig. 6C). A single mammalian-wide interspersed repeat c (MIRc) was found within the koala and opossum CGI. The sequence adjacent to the CGI was rich in transposable elements with lineage-specific differences in the repeat element composition. In mice and humans there was a prominent contribution of long tandem repeats (LTRs) to the sequence, monotremes had a dense arrangement of L2 long interspersed nuclear elements (LINES) and short interspersed nuclear elements (SINEs). In marsupials the sequence that *lncPRKACB* was transcribed from was comprised of SINEs, LINEs/L2 and other LINEs.

### Eutherian-specific *GNAS* structure

**Fig. 7:**
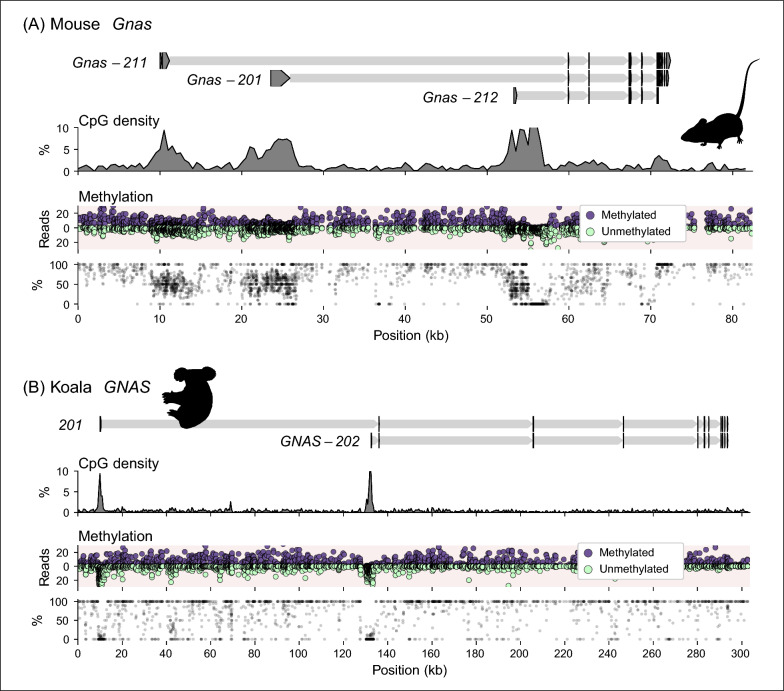
Marsupial-specific *GNAS* CGI structure. Scale illustration of the **A** mouse *Gnas* locus and the **B** koala *GNAS* locus, exons are arrowheads indicating the direction of transcription, introns are thinner shaded regions. CpG density is plotted as a percentage averaged over 500 bp windows. Methylated (purple) and unmethylated (green) WGBS read counts and the percent methylation are plotted over the region

The WGBS data sets were examined for further evidence of whether *PRKACB* imprinting might be marsupial-specific and *GNAS* imprinting eutherian-specific. The *Prkacb* locus in the mice WGBS data set (Fig. 2A) showed the CGI was largely unmethylated, with a methylation level of 9.2% (out of 1,106 reads). The *Gnas* locus is known to be imprinted in mice and functions in the same signaling pathway as the gene product for *Prkacb* [74]. The three CGIs at the mouse *Gnas* locus (Fig. 7A) were differentially methylated with methylation levels of 51.4% (3,811 reads), 52.4% (4,073 reads) and 33.9% (2,643 reads). The two CGIs at the koala *GNAS* locus (Fig. 7B) were unmethylated with methylation levels of 1.5% (2,382 reads) and 0.9% (1,818 reads).

## Discussion

**Fig. 8:**
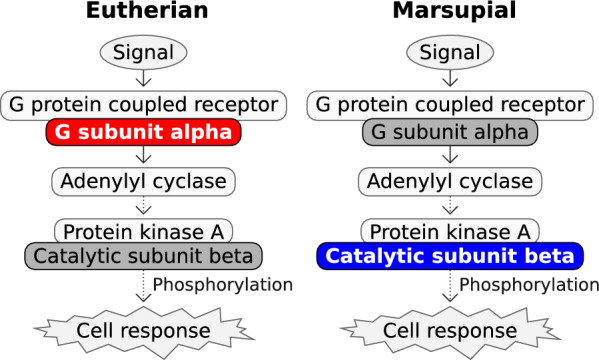
Lineage-specific imprinting of the cAMP/PKA signaling pathway. Simplified diagram of the cAMP/PKA signaling pathway indicating steps of interest subjected to lineage-specific imprinting. In eutherians (left), the *GNAS* gene is imprinted and *Gsα* (G subunit alpha) has expression from the maternal allele (red). In marsupials (right), the *PRKACB* gene is imprinted and is expressed from the paternal allele (blue). Dark grey indicates an unconfirmed or unknown imprinting status of a particular pathway step in the lineages

The *PRKACB* gene is imprinted in marsupials and is potentially a marsupial-specific imprinted gene. To our knowledge *PRKACB* has not been identified as an imprinted gene in any eutherian species. *PRKACB* imprinting could have been acquired specifically in the marsupial lineage. Maternal-specific methylation in the tammar and brushtail possum, and the differential methylation observed in the koala, suggest that *PRKACB* is imprinted in at least the Australian marsupials. Expression of an antisense *lncPRKACB* transcript and a long CGI in the grey short-tailed opossum raise the possibility that imprinting of *PRKACB* evolved early in the marsupial lineage. The imprinting of *PRKACB* in marsupials and *GNAS* in eutherians could indicate a conserved evolutionary pressure for imprinting of the cAMP/PKA signaling pathway with the lineages adapting by imprinting different genes in this pathway (Fig. 8).

Most known DMRs are methylated on the maternal allele but at least four DMRs are paternally methylated in eutherians [3, 104]. In mice CpG content was 5.6% at maternally methylated CGIs and 2.7% at paternally methylated CGIs [45], this could lead to imprint detection pipelines enriching for maternally methylated CGIs. Maternally and paternally methylated DMRs also have different locations. Paternal imprints are located in intergenic sequences while maternal imprints are typically found within genes. This correlates with global differences in the patterns of methylation acquisition during spermatogenesis and oogenesis [44, 104]. Here the known paternal imprint *H19* was not detected by our pipeline, although the other known paternal imprint *GPX7* was detected. Marsupial-specific paternal methylation of *GPX7* occurs at an intragenic CGI at the 3′ end of the gene [8]. The identification of more imprinted sites in marsupials could provide new information on the evolution of imprinted genes and their regulation.

We found multiple transcripts produced from the marsupial *PRKACB* locus including an imprinted *lncPRKACB* transcript and *PRKACB* isoform that both have start sites within the *PRKACB* DMR. In humans there are multiple, functionally non-redundant, isoforms of *PRKACB* and *PRKACA* [93]. The human *PRKACB* gene produces at least six splice variants due to alternative use of four 5′ exons [67, 92, 102]. The *PRKACB_iso1* transcript and the non-imprinted *PRKACB*_*iso2* transcript in the tammar and brushtail possum may correspond to the previously observed PKA Cα1/Cβ1 and CαL/Cβ2 isoforms [92]. In the brushtail possum biallelic expression of *PRKACB*_*iso2* in the liver appeared to correlate with the absence of *lncPRKACB* detection in this tissue. Isoform and tissue-specific paternal expression of *PRKACB* could be biologically significant in terms of gene regulation and the type and location of the cellular signals that are affected by a particular transcript.

Imprinting of *PRKACB* in marsupials and *GNAS* in eutherians might be different means of achieving similar physiological outcomes. Aberrant function of either *PRKACB* and *GNAS* contributes to pathological cAMP/PKA signalling in endocrine tumours [7, 20, 31, 52, 84]. Mutual exclusivity of mutations in *PRKACB* and *GNAS* suggests an epistatic relationship between these two genes. Alterations to *PRKACB* function through gene fusion are mutually exclusive from *GNAS* mutation in biliary tract cancer [61] and either mutually exclusive in pancreatic cancer [89] or not completely mutually exclusive [38]. Mutations in *PRKACA* (the *PRKACB* paralogue) and *GNAS* are mutually exclusive in Cushing’s syndrome and result in similarly sized adrenocortical adenoma diameter and similar increases in serum cortisol [84]. Lineage-specific imprinting of different steps in the cAMP/PKA pathway implies an evolutionary selection pressure applied at the level of the cell signaling pathway.

Several evolutionary theories have been proposed for genomic imprinting, with no one model well suited for describing all imprinted loci [94]. Under the parental conflict model for the selection of imprinting, paternally expressed genes are expected to promote offspring growth [26]. Increased *PRKACB* levels in papillary thyroid cancer cells [108] and adenoma of the pituitary gland [20] suggest that *PRKACB* influences offspring growth during development through effects on the endocrine glands. In general, *PRKACB* activity has been positively correlated with the proliferation and growth of cancer cells [18–20, 60]. There is a negative correlation between PRKACB levels and cell growth in non-small cell lung cancer tissue and transfection of *PRKACB* into LTEP-A2 cells decreased cell proliferation [11]. Whether *PRKACB* promotes cellular growth could depend on the specific tissue or other factors in the cellular signaling environment.

It is unclear what mechanism resulted in the expanded *PRKACB* CGI in marsupials that was associated with parent-of-origin-specific methylation. There was a lack of tandem repeats and transposable elements within the marsupial *PRKACB* CGI. The regions flanking the CGI were rich in transposable elements which could have allowed the CGI to have been inserted at the *PRKACB* start site from elsewhere. An important step in the generation of new imprinted regions is the insertion of DNA sequences which can be acquired from retroviruses or other unknown origins, or be duplicated by retroposition [40, 97]. The *lncPRKACB* transcript contains sequence derived from transposable elements and could be derived from retroviruses. The different sequence composition in eutherians suggests that *lncPRKACB* is found only in marsupials.

Limited studies are available addressing roles for *PRKACB* specifically in marsupials. In the Virginia opossum*, Didelphis virginiana, PRKACB* was upregulated in depigmented skin at the tip of the ear suggesting a function for PRKACB in melanocytes and phenotypic variation in marsupials [64]. Other marsupial studies suggest a role for cAMP/PKA signalling in reproductive function. The tammar relaxin peptide stimulated cAMP signaling in a relaxin-receptor expressing cell line [4]. The androgen metabolite 5α-androstane-3α, 17β-diol (5α-diol) mediates formation of the male urogenital tract in the tammar wallaby [86]. In human prostate cancer cells, 5α-diol stimulated the accumulation of intracellular cAMP and reduced *PRKACB* expression [65]. Signaling through the cAMP/PKA pathway occurs in marsupial sperm and has been associated with the capacitation process [5, 87].

Further components of the cAMP/PKA signaling pathway are imprinted in eutherians. Endothelin 3 (*Edn3*) is a vasoactive signaling peptide that acts via the cAMP/PKA pathway and is imprinted in the mouse brain [2, 9]. There are imprinted membrane receptors in humans and mice, including G protein-coupled receptor 1 (*GPR1/Gpr1*) and the calcitonin receptor (*CALCR/Caclr*), that signal through the cAMP/PKA pathway [15, 33, 46, 58]. The G protein-coupled receptor 78 (*GPR78*) and calcium voltage-gated channel subunit alpha 1C (*CACNA1C)* have placenta-specific DMRs in humans [81]. Regulators of intracellular cAMP concentration, phosphodiesterase 4D (*Pde4D*) and 10A (*Pde10A*) are imprinted in mice [34, 107]. The cAMP-dependent protein kinase inhibitor alpha (*PKIA*) is a strong candidate paternally-expressed gene in humans [27]. Downstream, the well-studied imprinted gene *KCNQ1* (potassium voltage-gated channel subfamily Q member 1) encodes a protein that is regulated by PKA-mediated phosphorylation [49].

Imprinted genes are most often studied in isolation or in terms of their respective clusters, but it was noted early on that multiple imprinted genes shared a common function in the insulin and insulin-like growth factor pathway [69]. The *IGF2R*, insulin-like growth factor 2 (*IGF2*) and insulin (*INS*) genes are imprinted in marsupials, but imprinting of growth factor receptor bound protein 10 (*GRB10*) is eutherian specific [63, 95, 96]. Imprinted genes have enriched gene ontology for organ development in humans and cation transport and G-protein signaling in mice [28]. The mouse imprinted gene network is enriched for extracellular matrix genes and regulates cell proliferation and differentiation [1]. The proteins encoded by human imprinted genes are connected to prominent hubs in the human interactome network, but do not have a high number of interactions themselves [82]. An imprinted transcription factor, pleiomorphic adenoma gene 1 like zinc finger 1 (*Plagl1*, also called *Zac1*), regulates a network of imprinted genes in eutherians, including *Gnas* [106]. *PLAGL1* was biallelically expressed in two opossum embryos [13] and in opossum and platypus lacks the CGI that is differentially methylated in eutherians [99] suggesting that *PLAGL1* is not an imprinted regulator of imprinted gene networks in marsupials.

The *GNAS* locus is transcriptionally complex. In humans 51 transcripts have been identified from the *GNAS* (NCBI gene ID: 2778) locus [73]. In mice the transcripts from the *Gnas* locus are regulated by three DMRs and have been summarised into six transcriptional units; the maternally-expressed *Nesp* and *F7*; the paternally-expressed *Exon 1A*, *Nespas* and *Gnasxl*; and *Gnas* which is maternally-expressed in certain cell types but biallelically-expressed in most tissues [32]. The alternative extra-large form of G_s_α, XL_s_α, encoded by *Gnasxl*, has identical cAMP signalling characteristics to G_s_α but is paternally expressed and has a more restricted expression pattern in neural and endocrine tissues [74]. If imprinting of *PRKACB* in marsupials is performing a similar function to *GNAS* imprinting in eutherians, it is not clear which transcript from the *GNAS* locus (e.g. *G*_*s*_*α* or *XL*_*s*_*α*) relates most directly in function to *PRKACB*.

Imprinting of *PRKACB* in marsupials and *GNAS* in eutherians is an example of convergent evolution indicating a conserved selection pressure for imprinting of the protein kinase A signaling pathway in therians with the two lineages adapting by imprinting different genes. This work further adds to the understanding that genomic imprinting is not fixed over evolutionary distance and affects different gene sets in divergent mammalian groups. Further examination will be required to uncover a deeper appreciation as to why marsupial and eutherian mammals possess unique sets of imprinted genes and how this relates to their respective reproductive strategies.

## Methods

### Whole-genome bisulfite sequencing (WGBS) data

A koala, *Phascolarctos cinereus*, female brain methylome was analysed using published WGBS data: SRX8207654 [88]. A mouse brain methylome was also analysed using a published WGBS dataset: SRX314948 [56] using GRCm39 (GCA_000001635.9) as a reference. The raw data was downloaded from the NCBI SRA (https://www.ncbi.nlm.nih.gov/sra). All WGBS-seq reads were trimmed using TrimGalore! v0.6.5 (https://www.github.com/FelixKrueger/TrimGalore) with clip_r1 and clip_r2 set to remove 8 bp. The trimmed reads were aligned to bisulfite converted versions of the koala genome (phaCin_unsw_v4.1, GCF_002099425.1) using Bowtie 2 v2.3.5.1 [51]. Aligned reads were then de-duplicated with the de-duplicate_bismark function in Bismark v0.22.3 [47]. CpG methylation was specifically called from the de-duplicated output using Bismark_methylation_extractor function in Bismark v0.22.3 with the bedGraph and report parameters.

### Methylation status at CpG islands

The contig-level koala DNA sequence, gene annotation, and methylation coverage was broken into 1 mb “windows”, to allow parallel computation. Within each window, CGIs were defined as 1 kb regions with over 3% CpG content located adjacent to a region meeting the same criteria. Methylation coverage was mapped to each CGI by genomic location and averaged over the CpG sites within each region. CGIs were associated with an annotated gene feature by being within the start and end of the feature, starting inside the feature, ending inside the feature, spanning the start and end of the feature, being within 5 kb of the feature start site, or being within 5 kb downstream of the feature end.

CGIs were identified as candidate DMRs using stringent criteria in order to reduce the size of the candidate list for subsequent testing. The criteria used were based on WGBS signal at the koala *IGF2R* DMR and included being 45–55% methylated, having a standard deviation of methylation (between CpG sites within the CGI) of less than 25%, having over 400 sequencing reads, having over 95 CpG sites and having over 4% CpG content. The scripts required to reproduce the processed data are available on GitHub (https://www.github.com/trentnewman/dmrcan). DNA methylation in the context of gene features was assessed in SeqMonk v1.47.2 (https://www.bioinformatics.babraham.ac.uk/projects/seqmonk/). Gene diagrams were prepared with the DNA Features Viewer [111]. When plotting WGBS methylation calls across a gene locus, including the CGI and non-CGI sites, the Bismark coverage file was used.

### Animals

Tammar wallabies, *Notomacropus (Macropus) eugenii*, originating from Kangaroo Island, South Australia, were held in open grassy yards in our breeding colony at the University of Melbourne. Animal experiments were approved by the University of Melbourne Animal Experimentation Ethics Committees and followed the Australian National Health and Medical Research Council [62] guidelines. Brushtail possum (*Trichosurus vulpecula*) tissues were sourced from freshly deceased animals killed as part of a pest-control programme in New Zealand [8].

Tammar differential methylation was assessed in pouch young (PY) tail tissue from four animals (out of twelve that were screened for SNPs) that were sampled between day 73 and 81 post-partum (pp), with the parental origins of expression determined by genotyping samples from the maternal liver. Whole RNA long-read transcriptomes were generated from ovary and testis samples taken at day 148 pp (PRJNA1145609). Tammar allele-specific transcription of the lncRNA was assessed in PY liver tissues from ten animals (out of eleven that were screened) between day 35 and 41 pp and PY rhomboid skeletal muscle from four animals (out of six that were screened) between day 18 and 25 pp, matched to samples of the maternal liver. Brushtail possum differential methylation was assessed in PY tail tissue from two animals (out of five that were screened for SNPs) that were sampled between days 18 and 99 pp, with the parental origins determined by genotyping samples from the maternal liver. Brushtail possum allele-specific transcription of the lncRNA was assessed in PY muscle from one day 22 pp animal, and PY liver from three animals between days 22 and 120 pp, with the parental origins determined by genotyping samples from the maternal liver. Allele-specific expression of the *PRKACB* coding gene was assessed in PY liver from four animals (out of five that were screened for SNPs) and PY muscle from two animals that were sampled between days 12 and 99 pp, with the parental origins determined by genotyping samples from the maternal liver. Tissue samples were collected as described previously [8, 36, 98], snap frozen in liquid nitrogen and stored at -80 °C prior to use.

### Genomic DNA extraction

Maternal and PY tammar genomic DNA (gDNA) was prepared from frozen tissue using the Wizard Genomic DNA Purification Kit (cat. no. A1120, Promega, Madison, WI, USA) with a T10 basic handheld homogenizer (IKA, Staufen, Germany). Genotyping the candidate DMR was performed by PCR with GoTaq Master Mix (cat. no. M5123, Promega, Madison, WI, USA) and the genotyping primers indicated (Additional File 1). The products were gel purified and sequenced by the Australian Genome Research Facility (AGRF). DNA was extracted from brushtail possum tissue following protocols described in [8] (see DNA extraction for all other analyses).

### Bisulfite clone sequencing

Bisulfite-converted DNA for methylation analysis was prepared from 1 μg of tammar genomic DNA using the EpiMark Bisulfite Conversion Kit (cat. no. E33185, New England Biolabs, Ipswich, MA, USA). Bisulfite primers (see Additional file 1) were designed using MethPrimer [54]. PCR of bisulfite-treated gDNA was performed using EpiTaq HS (cat. no. R110A, TaKaRa, Kusatsu, Japan). PCR products were purified using the QIAquick Gel Extraction Kit (cat. no. 28706, Qiagen, Venlo, Netherlands). Purified bisulfite PCR products were ligated into the pGEM-T easy vector cloning system (cat. no. A1360, Promega, Madison, WI, USA). JM109 competent cells (cat. no. L2001, Promega, Madison, WI, USA) were transformed with the ligation products and plated onto LB/Amp/IPTG/X-Gal. Plasmids were prepared with the Wizard Plus SV Minipreps DNA Purification System (cat. no. A1460, Promega, Madison, WI, USA) and sequenced by AGRF (using the M13 reverse primer, Additional file 1). The Sanger sequencing results from individual clones were genotyped as being either maternal or paternal. The sequencing reads were trimmed for vector sequence and entered into QUMA [48] to call CpG methylation status.

### Allele-specific methylation sequencing

A dual-indexing, four-primer PCR-based assay was used for bisulfite amplicon sequencing in brushtail possum [16], with the following modifications. Bisulfite primers (see Additional file 1) were designed using MethPrimer [54], with the addition of a “handle” sequence such that during amplification the handle is incorporated into the amplicon. DNA (500 ng) was bisulfite converted using the EZ-96 DNA Methylation MagPrep kit (cat. no. D5040, Zymo Research, Irvine, CA, USA), according to the manufacturer’s guidelines. Following the first round of PCR amplification KAPA HiFi Uracil + ReadyMix (cat. no. KK2801, Roche, Basel, Switzerland), the PCR reactions were cleaned up and size-selected using a 0.9 × SPRI beads (cat. no. B23317, Beckman Coulter, Brea, CA, USA) diluted in standard PEG buffer [66]. The DNA was eluted in nuclease-free water and used as a template in a second round of PCR amplification, with primers complementary to the handle and an overhang consisting of a multiplex sequencing index and Illumina adapters. The final amplicons were pooled in equivolume amounts, cleaned-up and size-selected using a 0.9 × SPRI beads diluted in standard PEG buffer and sequenced on the iSeq100 (Illumina, San Diego, CA, USA) to generate 150 bp paired-end reads. Paired reads were trimmed using TrimGalore! v0.6.7 in a two-step process: first, to remove adapters, and second, to remove low-quality base calls (Phred score < 20). Reads with a different allele at the SNP of interest within the amplicon were extracted into separate files using grep. Separated reads were mapped to the original, unconverted DNA sequence using bwameth v0.2.6 [70] and methylation calls were extracted using MethylDackel v0.5.2 (https://www.github.com/dpryan79/MethylDackel).

### Transcriptomes

RNA was prepared from frozen tissue using the GenElute Mammalian Total RNA Miniprep Kit (cat. no. RTN70-1KT, Sigma-Aldrich, St. Louis, MO, USA). RNA quality and integrity was assessed using an Agilent TapeStation 2200, only high-quality RNA samples with a RIN value over 8 were used.

Long-read PCR-cDNA libraries were prepared with the barcoding kit (SQK-PCB109, Oxford Nanopore Technologies, Oxford, United Kingdom) starting from 100 ng total RNA and with 16 cycles of PCR and 2 min 30 s of extension time. 50 ng of a pool of 12 barcodes with sequencing adapters were loaded onto one PromethION flow cell (FLO-PRO002, R9.4.1), and run on a PromethION P24 (MinKNOW 21.10.8). The Reads, which were basecalled and demultiplexed using guppy 5.0.17 in “super-accurate” mode, without adapter trimming. Pass reads (q > 10) were aligned to the tammar genome using minimap2 [53]. Restrander [85] was used to orient the reads according to the original RNA strand.

Publicly available tammar adult ovary (DRX012254) and testis (DRX012262) RNA-seq data sets were analysed. The paired-end reads were trimmed using TrimGalore! v0.6.10 (https://github.com/FelixKrueger/TrimGalore), aligned to the tammar wallaby genome (see: GCA_028372415.1), using HISAT2 v2.2.1 [43] with the "–rna-strandness RF" option and mapped reads assigned to each strand with Samtools v1.16.1 [55]. Publicly available grey short-tailed opossum ovary (SRX149633), testis (SRX149630) and muscle (SRX149626) RNA-seq data was analysed as above but aligning to the opossum reference genome (see: GCF_027887165.1). Brushtail possum PY muscle (SRR22399473), spleen (SRR22399467) and liver (SRR22399480) RNA-seq data sets were analysed. The single-end reads were trimmed, then aligned to the brushtail possum genome (GCF_011100635.1) with the "–rna-strandness R" option. Transcript structures were generated using Stringtie with the "-L" in the case of long-reads and merged for each species with an "-F 0.5" [71]. Transcript abundance was estimated using the Stringtie Ballgown FPKM output of fragments per kilobase of transcript per million mapped reads. Sashimi lines indicating intron-spanning reads were prepared using the RegTools v1 junctions extract tool (https://github.com/griffithlab/regtools).

### Allele-specific expression

PY RNA was prepared from snap frozen tissue using the GenElute Mammalian Total RNA Miniprep Kit (cat. no. RTN70-1KT, Sigma-Aldrich, St. Louis, MO, USA). cDNA was prepared from 1 μg of RNA using the Superscript IV First-Strand Synthesis System (cat. no. 18091050, ThermoFisher Scientific, Waltham, MA, USA), primed with oligo(dT)_20_. Allele-specific expression analysis was performed by RT-PCR using the expression primers listed (Additional file 1) with the cDNA, above. PCR products were gel extracted using the QIAquick Gel Extraction Kit (cat. no. 28706, Qiagen, Venlo, Netherlands) and sent for Sanger sequencing by AGRF. Allele-specific expression of brushtail possum RNA was performed following protocols described in [8] (see RNA extraction and preparation for all other analyses; cDNA synthesis and reverse transcription-PCR (RT-PCR) amplification).

### *PRKACB* species comparison

The genomic DNA sequence for the region containing the *TTLL7, PRKACB* and *SAMD13* genes was taken from the human (GRCh38.p14, GCA_000001405.29), mouse (GRCm39, GCA_000001635.9), elephant (mLoxAfr1.hap2, GCA_030014295.1), echidna (mTacAcu1.pri, GCA_015852505.1), platypus (mOrnAna1.pri.v4, GCA_004115215.4), agile gracile opossum (AgileGrace, GCA_016433145.1), grey short-tailed opossum (mMonDom1.pri, GCA_027887165.1), koala (phaCin_unsw_v4.1, GCA_002099425.1), tammar (mMacEug1.pri, GCA_028372415.1) and brushtail possum (mTriVul1.pri, GCA_011100635.1) genomes. The transcript structures assembled above informed the position of the tammar and brushtail possum *PRKACB* gene start site. The mouse, elephant, platypus, agile gracile opossum, grey short-tailed opossum and brushtail possum sequences were reversed to match the orientation of the other species.

Synteny was compared by pairwise species alignment of the DNA in the *PRKACB* gene region, between the *TTLL7* and *SAMD13* genes, using LASTZ v1.04.15 [30], https://github.com/lastz/lastz). High-sensitivity alignment was performed using the “–transition”, “–step = 1”, “–gfextend”, “–chain”, “–gapped” and “–strand = both” options. For a comparison of the *PRKACB* start site between species the DNA sequence ± 25 kb was assessed. Transposable elements adjacent to the *PRKACB* start site were detected using RepeatMasker v4.1.5 [101] with the "-species mammals" option. Tandem repeats were detected using Tandem Repeats Finder v4.09.1 [6] (https://github.com/Benson-Genomics-Lab/TRF with the recommended parameters. The scripts required to reproduce the synteny tracks are available on GitHub (https://www.github.com/trentnewman/syntrk).

## Supplementary Information

**Additional file 1.** Primers used in this study. "Geno": genotyping primers, "BS": primers for BS-converted DNA, “Expr”: primers used for expression analysis. The lower case oligos indicate the “handle” used for sequencing. Primers starting “Me” are for tammar wallaby, primers starting “Tv” are for brushtail possum.

## Data Availability

The datasets analysed during the current study are available on the National Center for Biotechnology Information (NCBI) repository: https://www.ncbi.nlm.nih.gov/.

## Abbreviations

cAMP: Cyclic adenosine 3′, 5′-monophosphate
CGI: CpG island
CpG: Cytosine guanine dinucleotide
DMR: Differentially methylated region
FPKM: Fragments per kilobase of transcript per million mapped reads
GNAS: Guanine nucleotide-binding protein alpha subunit
Gsα: G-protein alpha-subunit
IGF2R: Insulin-like growth factor 2 receptor
lncRNA: Long noncoding RNA
PKA: Protein kinase A
pp: Post-partum
PRKACB: Protein kinase A catalytic subunit beta
PY: Pouch young
WGBS: Whole genome bisulfite sequencing
